# Spontaneous Pneumomediastinum Following Intense Gym Exercise in a Healthy Young Male: A Case Report

**DOI:** 10.7759/cureus.104100

**Published:** 2026-02-23

**Authors:** Helmy M Elhag, Amr Elmoheen, Omar Khyatt, M. Shakeeb, Nood Dhafi R Al-Marri

**Affiliations:** 1 Emergency Medicine, Hamad Medical Corporation, Doha, QAT; 2 Emergency Medicine, Qatar University, Doha, QAT; 3 Radiology, Alwakra Hospital, Hamad Medical Corporation, Doha, QAT

**Keywords:** exercise induced pneumomediastinum, macklin effect, spontaneous pneumomediastinum, subcutaneous emphysema, weightlifting injury, young adult

## Abstract

Spontaneous pneumomediastinum (SPM) is a rare and usually self-limiting condition that may occur in young and otherwise healthy individuals, often after activities that cause a sudden rise in intrathoracic pressure. We present the case of a 21-year-old man who developed acute throat pain and difficulty swallowing immediately following intense weightlifting. Physical examination was suggestive of cervical subcutaneous emphysema, and imaging confirmed pneumomediastinum without evidence of esophageal or airway injury. The patient was managed conservatively with rest, analgesics, supplemental oxygen, and observation, resulting in rapid symptom improvement and complete radiologic resolution. This case highlights the need to consider SPM in patients presenting with chest or neck symptoms after strenuous physical activity, as early recognition supports appropriate conservative management and helps avoid unnecessary invasive interventions.

## Introduction

Spontaneous pneumomediastinum (SPM) refers to the presence of air within the mediastinum without an identifiable traumatic or iatrogenic cause. Exercise-related spontaneous pneumothorax is rare, and this particular case illustrates a classic exertional presentation (heavy lifting, intense gym workout) with prominent throat symptoms, with rapid improvement and complete radiographic resolution on conservative management [1,2]. Its incidence is low, with only a few cases reported per ten thousand hospital or emergency department admissions. SPM occurs more frequently in adolescent and young adult males and is often associated with events that cause a sudden rise in intrathoracic pressure [3]. The most widely accepted explanation for its development is the Macklin effect, in which an abrupt increase in intra-alveolar pressure leads to alveolar rupture, allowing air to track along peribronchovascular sheaths into the mediastinum [4]. This process may be triggered by activities such as intense physical exertion, coughing, vomiting, or other Valsalva maneuvers [3,5]. Clinically, SPM often presents with nonspecific symptoms, including chest or neck pain, dysphagia or odynophagia, and subcutaneous emphysema, which can closely resemble more serious conditions. Although both conditions can manifest with acute chest pain, pneumomediastinum is the presence of air in the mediastinum, while pneumothorax is the presence of air in the pleural space and can lead to lung collapse. Primary spontaneous pneumothorax tends to occur in tall, thin young males and is associated with smoking, while secondary pneumothorax is associated with underlying lung disease. Understanding these differences allows for appropriate urgency, surveillance, and imaging, as acute management may be altered if tension physiology occurs, and careful assessment of the lung apices on initial and follow-up imaging is required [6]. Because of this overlap with potentially life-threatening diagnoses such as Boerhaave’s syndrome or pulmonary embolism, careful clinical assessment and appropriate imaging are essential [3,5]. While chest radiography can often identify mediastinal air, computed tomography provides greater sensitivity and helps exclude alternative causes [3,4]. In this report, we present a rare case of SPM in a healthy young man following intense physical exertion, highlighting the diagnostic approach and successful conservative management, along with a brief review of the literature.

## Case presentation

A 21-year-old male college student with no significant past medical history presented to the emergency department with sudden-onset lower throat pain and difficulty swallowing that began immediately after a heavy weightlifting session at the gym. He explained that near the end of his workout, while straining during a maximal-effort lift, he experienced a sharp pain in the lower neck and upper chest, followed by discomfort with swallowing. He denied any trauma to the neck or chest, recent upper respiratory symptoms, coughing, vomiting, or ingestion of a foreign body. He was a non-smoker and reported no use of illicit drugs or vaping. There was also no history of asthma or chronic lung disease.

On physical examination, the patient appeared anxious but was clinically stable. His vital signs were within normal limits, including a blood pressure of 122/78 mmHg, heart rate of 88 beats per minute, respiratory rate of 18 breaths per minute, oxygen saturation of 98% on room air, and an oral temperature of 36.8 °C. Mild swelling was noted in the supraclavicular region, with palpable crepitus extending into the lower neck, consistent with subcutaneous emphysema. There was no point tenderness of the chest wall or pectoral area, no erythema or bruising evident, and the patient did not complain of a ‘giving way’ sensation during the lift, suggestive of acute muscle tear. Cardiac examination revealed normal heart sounds without murmurs or pericardial crunching sounds, and Hamman’s sign was absent. Lung auscultation was clear bilaterally, with no wheezes or crackles, and there was no stridor or tracheal deviation. The remainder of the examination was unremarkable, including a non-tender chest wall and a normal abdominal examination.

In view of the localized throat pain and subcutaneous emphysema, an otolaryngology (ENT) consultation was obtained to assess for possible pharyngeal or laryngeal injury. Flexible fiber-optic nasolaryngoscopy revealed no evidence of mucosal injury, vocal cord abnormalities, or other upper airway pathology. Laboratory studies, including a complete blood count and basic metabolic panel, were within normal limits, with only a minimal elevation in C-reactive protein (8 mg/L). An electrocardiogram demonstrated a normal sinus rhythm without any ischemic changes.

**Figure 1:**
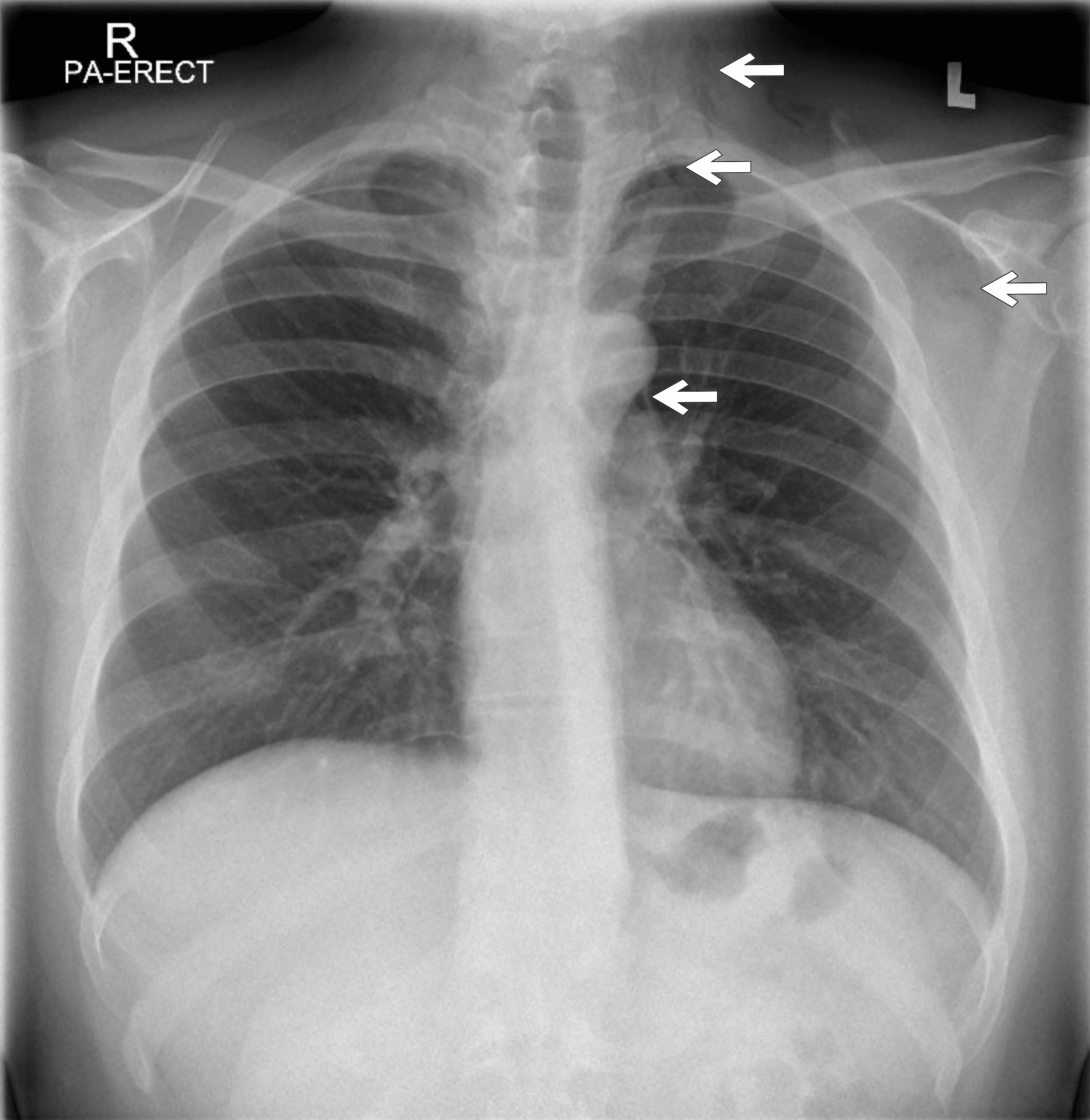
Posteroanterior (PA) chest radiograph showing linear radiolucent streaks along the left side of the neck, adjacent to the aortic knuckle, extending into the soft tissues of the left axilla (white arrows). These findings are consistent with pneumomediastinum associated with subcutaneous (soft tissue) emphysema.

An initial chest radiograph, including posteroanterior and lateral views, was performed as the first imaging study. This revealed streaky areas of radiolucency along the mediastinum and at the base of the neck, findings consistent with mediastinal emphysema, along with subtle subcutaneous emphysema in the cervical region. The lung fields were clear, with no evidence of pneumothorax on the X-ray (Figure 1).

**Figure 2:**
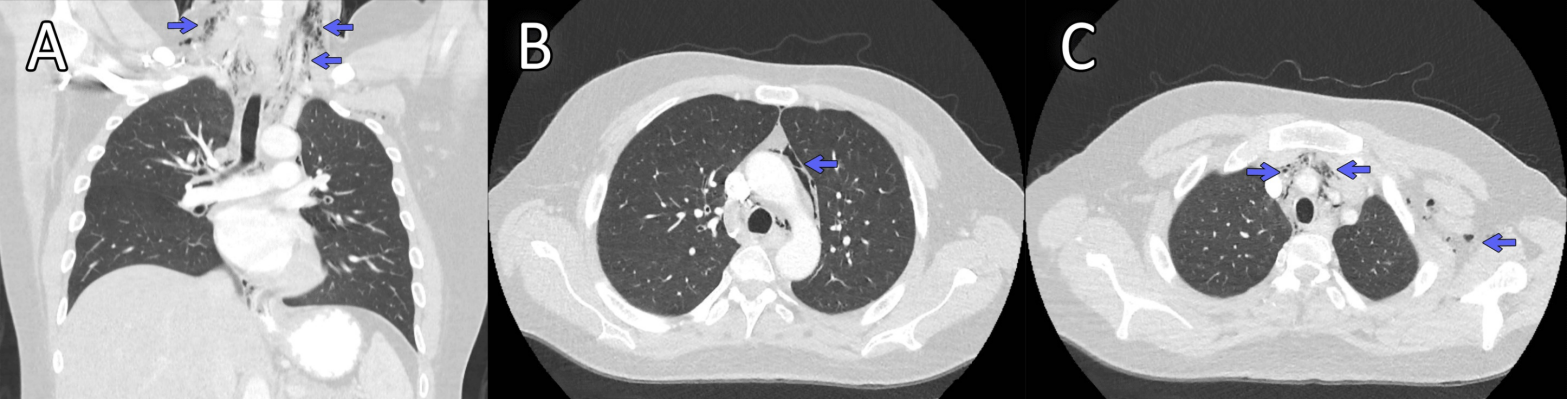
Computed tomography (CT) of the chest. CT of the chest in lung window settings, demonstrating the following findings: (A) Coronal section showing linear air streaks tracking along the right and left cervical soft tissues (blue arrows). (B) Axial section at the level of the aortic arch demonstrating free air outlining the aortic arch (blue arrows). (C) Axial section of the upper chest showing air streaks within the anterior mediastinum, extending along the major mediastinal vessels into the soft tissues of the left axilla (blue arrows). Overall, these findings are consistent with pneumomediastinum associated with subcutaneous (soft tissue) emphysema.

In view of the combination of odynophagia and cervical subcutaneous emphysema, a CT scan of the neck and chest was requested to confirm the diagnosis, assess the extent, and look for complications. The CT scan was done with IV contrast (no oral contrast); it revealed extensive pneumomediastinum with air tracking in the cervical region, and no CT signs of esophageal perforation (no wall defect, no mediastinal fluid/collection, and no contrast extravasation), and no pneumothorax or pleural effusion (Figure 2).

Taken together with the clinical findings, these imaging results confirmed the diagnosis of SPM. Acute coronary syndrome and acute pericarditis were felt to be unlikely due to the lack of characteristic ischemic or position-related chest pain, an unremarkable cardiovascular exam without pericardial rub, and a normal ECG without evidence of ischemia or pericarditis; cardiac enzymes were not indicative of myocardial injury. Esophageal spasm was entertained, but the presence of cervical subcutaneous emphysema and supporting imaging studies led to SPM as the integrating diagnosis.

**Figure 3:**
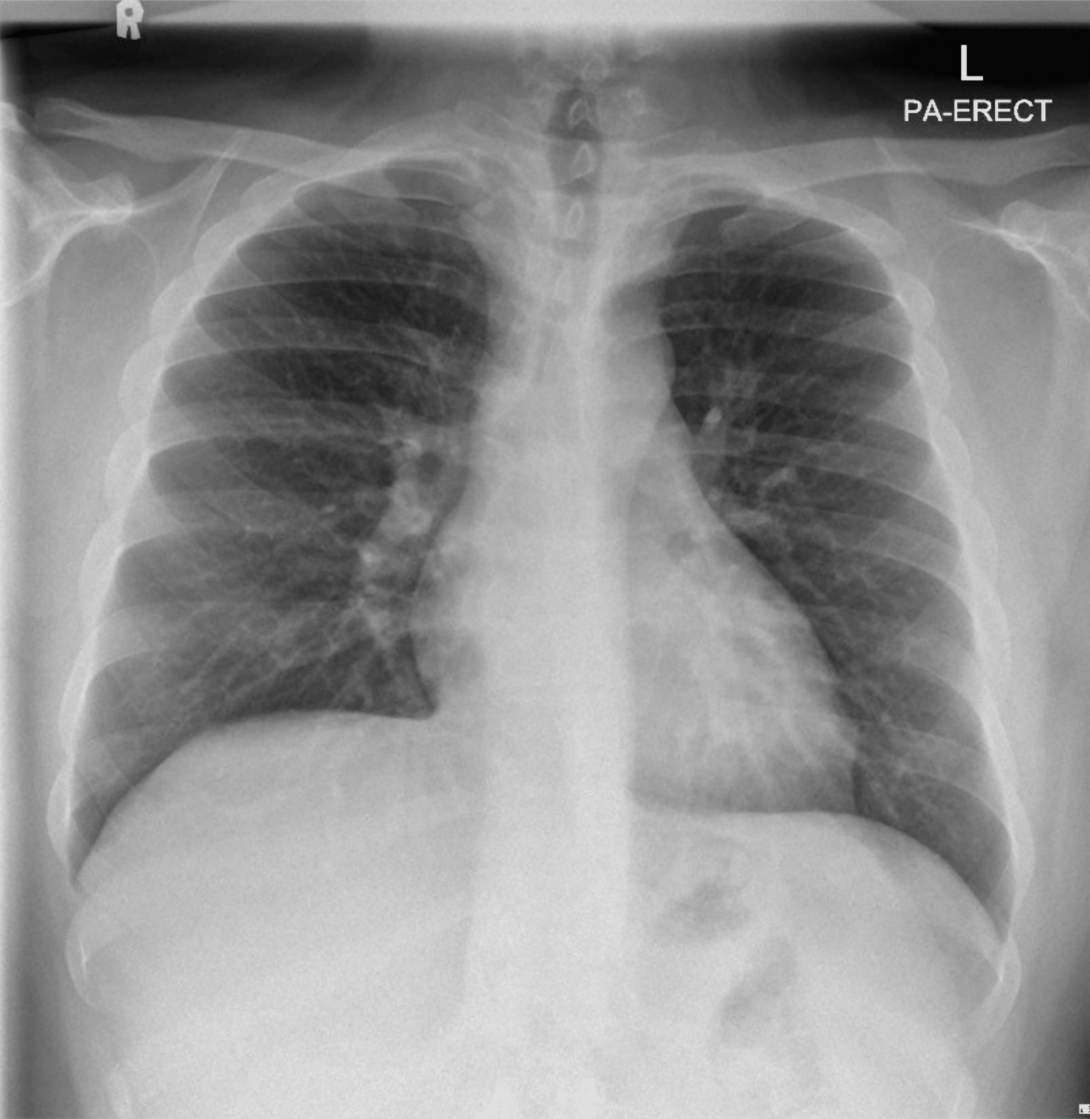
Repeat chest radiograph obtained at 48 hours demonstrating substantial resolution of the mediastinal air, correlating with clinical improvement.

The patient was admitted for observation because of the severity of the mediastinal/cervical air and for repeated examinations while on supplemental oxygen, with low but significant concern for complications. He was placed on bed rest with the head of the bed elevated and advised to avoid activities that could increase intrathoracic pressure, including strenuous exertion and Valsalva maneuvers. Supplemental oxygen was administered at 10 L/min via face mask to promote nitrogen washout and facilitate resorption of the mediastinal air. His pain was adequately controlled with acetaminophen and nonsteroidal anti-inflammatory drugs, and opioid analgesia was not required. As there was no indication of infection or esophageal injury, antibiotics were not prescribed. The serial assessments particularly focused on the following: vital signs, oxygen saturation, work of breathing, chest auscultation, pain severity, and the extent/progression of cervical subcutaneous crepitus, as well as red-flag symptoms, which include worsening dyspnea, severe chest pain, fever, and hemodynamic instability. The repeat chest radiograph at 48 hours showed a significant improvement in the mediastinal air (Figure 3).

Given his continued clinical improvement and stable condition, he was discharged on the third hospital day with instructions to rest and avoid heavy lifting, strenuous exercise, or forceful Valsalva-like activities for at least two weeks. At follow-up one week later, he remained asymptomatic, and a repeat chest X-ray confirmed complete resolution of the pneumomediastinum with no residual soft tissue air. The patient provided informed consent for publication of this case.

## Discussion

SPM is an uncommon condition, particularly in young, otherwise healthy individuals without pre-existing lung disease. Reported incidence rates range from approximately 1 in 7,000 to 1 in 25,000 hospital or emergency department admissions [5,7]. The condition predominantly affects young males, which is consistent with the demographic profile of our patient [1,7]. SPM is most often triggered by events that cause a sudden increase in intrathoracic pressure. Although asthma exacerbations and severe coughing are well-recognized precipitating factors, other triggers have been described, including inhalation of recreational drugs, forceful shouting, childbirth, and intense physical exertion [8,9]. In a large review of 535 cases of SPM, approximately 21% of patients reported recent vigorous exercise or heavy exertion, supporting the association seen in our patient following weightlifting [1].

The most widely accepted explanation for the development of SPM is the Macklin effect, first described in 1944. According to this mechanism, an abrupt rise in intra-alveolar pressure leads to alveolar rupture, allowing air to escape and dissect along peribronchovascular tissue planes into the mediastinum [8]. Activities that involve significant straining or Valsalva maneuvers, such as heavy lifting in a gym setting, can generate sufficient intrathoracic pressure to result in alveolar micro-rupture and subsequent pneumomediastinum [2,9].

Clinically, SPM presents with a range of symptoms that may include retrosternal chest pain, neck or throat discomfort, shortness of breath, and subcutaneous emphysema involving the neck or chest wall [5,7]. A classic but infrequently observed physical finding is Hamman’s sign, described as a crunching or crackling sound heard in synchrony with the heartbeat during auscultation; however, this sign is present in only about 20%-30% of cases [5]. In this case, the most informative clinical finding was the presence of subcutaneous emphysema in the neck, which, when combined with the clinical context, strongly suggested pneumomediastinum.

The differential diagnosis for acute chest or neck pain accompanied by mediastinal air must be approached carefully. Boerhaave’s syndrome, or esophageal rupture, is a critical consideration, as it can also present with chest pain and odynophagia and is associated with significant morbidity if not promptly treated [10]. Although the absence of vomiting and the patient’s stable condition made esophageal rupture unlikely in this case, it was appropriately excluded with imaging. Other important differential diagnoses include pneumothorax and pulmonary embolism, neither of which was supported by the imaging findings. It is also worth noting that up to 10% of patients with SPM may have a small concurrent pneumothorax, underscoring the importance of careful assessment of the lung apices on imaging [7]. In our patient, no pneumothorax was identified on either initial or follow-up studies.

Imaging plays a crucial role in the diagnosis of SPM and in excluding potentially life-threatening conditions. A chest X-ray is typically the first imaging study performed and is able to identify mediastinal emphysema in most cases [7]. However, small amounts of mediastinal air may not always be detected on plain radiographs. For this reason, computed tomography of the chest is considered the gold standard for confirming pneumomediastinum [8]. In uncomplicated cases of SPM, additional invasive investigations such as bronchoscopy or esophagogastroscopy are generally unnecessary and can be safely avoided [7]. In the absence of high-risk criteria (forceful vomiting, sepsis, pleural effusion, or mediastinal fluid), a negative CT scan significantly reduces the risk of esophageal perforation; hence, a further fluoroscopic esophagram was unnecessary in our stable patient.

The management of SPM is largely conservative, reflecting its typically self-limiting nature. In uncomplicated cases, treatment is focused on supportive care and observation [1,7]. High-flow supplemental oxygen may be administered to accelerate reabsorption of free air [8]. Most cases resolve within a few days to one week, and the rate of recurrence in SPM is low (~1%). The prognosis is generally excellent with nonoperative management. Regarding exertional SPM, patients are generally advised to avoid strenuous activity and Valsalva maneuvers for one to two weeks, followed by a gradual return to activity once symptoms have resolved and clinical reassessment is reassuring [2,9].

This case highlights the importance of considering SPM in patients presenting with unexplained chest or neck symptoms following intense physical activity. Early recognition and appropriate imaging allow accurate diagnosis, avoidance of unnecessary invasive procedures, and reassurance regarding the excellent prognosis [1,7].

## Conclusions

SPM is a relatively rare but significant consideration for acute chest or throat pain in young adults, especially following strenuous exercise such as weightlifting. After ruling out more immediately life-threatening diagnoses, such as esophageal perforation, most patients can be treated successfully with observation, pain control, and activity restriction. Greater awareness of this condition within the emergency department can help facilitate earlier diagnosis and prevent unnecessary invasive procedures.
